# Potential Prognostic Impact of High-Sensitivity C-Reactive Protein in the Analysis of Whole-Heart Myocardial Mechanics and Morphometry: Prospective CMR-Based Study

**DOI:** 10.1177/11795468251369240

**Published:** 2025-09-16

**Authors:** Karolina Melinyte-Ankudavice, Gabriele Jakuskaite, Gryte Galnaitiene, Gabriele Darge, Egle Ereminiene, Gintare Sakalyte, Jurgita Plisiene, Renaldas Jurkevicius

**Affiliations:** 1 Department of Cardiology, Medical Academy, Lithuanian University of Health Sciences, Kaunas, Lithuania; 2 Laboratory of Clinical Cardiology, Institute of Cardiology, Lithuanian University of Health Sciences, Kaunas, Lithuania; 3 Department of Radiology, Medical Academy, Lithuanian University of Health Sciences, Kaunas, Lithuania; 4 Faculty of Informatics, Vytautas Magnus University, Kaunas, Lithuania

**Keywords:** cardiac magnetic resonance, heart failure, non-ischemic dilated cardiomyopathy, biomarkers, high-sensitivity C-reactive protein

## Abstract

**Background:**

Non-ischemic dilated cardiomyopathy (NIDCM) remains a significant part of heart failure (HF) origin, requiring more detailed investigation of the whole heart. This study aimed to examine the commonly used biomarkers in clinical practice and their relationship with early alterations in whole-heart myocardial mechanics and morphometry in patients with NIDCM.

**Methods:**

In this prospective single-center study, 98 patients (mean age 49.5 ± 10.1 years; 69.4% male) were included in the final sample during the first phase, when the diagnosis of NIDCM was made. After 1 year, 42 patients were evaluated during the second follow-up phase. The cardiac magnetic resonance was used to analyze whole-heart myocardial mechanics and morphometry. Biomarkers (troponin I, C-reactive protein (CRP), high-sensitivity CRP (hs-CRP), brain natriuretic peptide, suppression of tumorigenicity 2, and neutrophil to lymphocyte were assessed at the time of the diagnosis.

**Results:**

The strongest correlations were observed between hs-CRP levels and left atrial (LA) global longitudinal strain (GLS) changes after 1 year (*r* = −.659, *P* < .001). It was revealed that the cut-off value of 3.6 mg/l of hs-CRP can prognosticate to find a reduced LA GLS with a sensitivity of 100% and specificity of 87% (AUC, 0.833; 95% CI, 0.65–1.008; *P* < .001).Other biomarkers had weaker associations with myocardial mechanics and morphometry; relationships were established only with left heart parameters.

**Conclusion:**

In NIDCM patients, the main biomarkers of HF are related to early changes in left-heart myocardial mechanics and morphometrics. The strongest relationship was between the initial levels of hs-CRP and early changes in LA GLS.

## Introduction

NIDCM is a serious and often under-recognized condition characterized by the enlargement and systolic dysfunction of the left or both ventricles.^1^ Unlike ischemic dilated cardiomyopathy, which is caused by coronary artery disease, NIDCM can result from various causes, many of which are not well understood.^2^

Initial changes in myocardial mechanics and morphometry in NIDCM are often the earliest indicators of structural and functional changes in the heart, frequently occurring before more noticeable symptoms of heart failure emerge.^2^ The progression of the disease can be gradual, especially in cases of familial or idiopathic dilated cardiomyopathy (DCM), and may go unnoticed for a considerable period before a diagnosis is made.^2,3^ Around 25% of DCM patients experiencing the recent onset of HF symptoms may see spontaneous improvement.^2,4^ However, those with symptoms lasting longer than 3 months and presenting with severe clinical decompensation typically have a lower chance of recovery.^2^

The changes, which occur at both the cellular and macroscopic levels, can be detected before overt clinical signs develop and may provide valuable insights into the progression of the disease. Advanced imaging techniques, such as cardiac magnetic resonance (CMR) imaging, echocardiography, and strain imaging, allow for detecting these alterations long before symptoms become clinically apparent.^5^ CMR has become an indispensable tool in evaluating NIDCM by providing detailed, non-invasive insights into both the structure and function of the heart.^5,6^ Feature tracking (FT) imaging, initially developed for echocardiography, is now validated for measuring left ventricular (LV) strain with CMR and offers potentially greater clinical value than relying on left ventricular ejection fraction (LVEF) measurements alone.^6^

The progression of heart failure is driven by various biological processes, including inflammation, oxidative stress, neurohormonal activation, and vascular remodeling.^7^ Identifying biomarkers associated with DCM is crucial for understanding diagnosis, prognosis, risk stratification, and treatment response. Previous studies have examined numerous potential biomarkers in patients with DCM or NIDCM. However, there is a lack of studies that identified associations between biomarkers and changes in whole-heart myocardial mechanics and morphometry.^8^

Despite the increasing research on this topic, most studies investigating NIDCM have primarily focused on changes in isolated CMR parameters.^6,9^ However, incorporating additional biomarkers may provide more insight into predicting outcomes in NIDCM. We aimed to investigate the most frequently used biomarkers in clinical practice linked to early changes in myocardial mechanics and the morphometrics of the whole heart, including both ventricles and atria, in patients with DCM.

## Methods

### Study Population

It is a prospective study that enrolled 110 patients with NIDCM. NIDCM was defined as an LV enlargement and global systolic function impairment (LVEF < 45%) in the absence of coronary artery disease or increased loading conditions (hypertension, valve disease, etc.). LV enlargement was defined by the latest Recommendations for Cardiac Chamber Quantification by Echocardiography in Adults.^10^

The patient was included in the study once the DCM phenotype was identified and ischemic heart disease was excluded. All participants underwent either invasive coronary angiography or coronary multislice computed tomography. Ischemic heart disease was defined by a history of myocardial infarction, revascularization procedures, or coronary artery stenosis greater than 50%. All participants were over 18 years of age.

Exclusion criteria:

■ Primary valvular heart disease.
■ Untreated or treatment-resistant arterial hypertension or using > 2 antihypertensive drugs.
■ Chronic kidney disease (> stage 3).
■ Inflammatory heart disease.
■ History of pulmonary embolism.
■ Diagnosed with peripartum cardiomyopathy.
■ Implanted cardiac devices.
■ HF caused by prolonged tachycardia.
■ Suspected toxic damage (alcohol, drugs).
■ Poor quality of CMR images.

### Study Phases

The study consisted of 2 phases:

- Patients were enrolled and examined for the first time during the initial phase of the study. Those diagnosed with NIDCM and who did not have chronic or worsening heart failure met the inclusion criteria. Out of 110 patients initially diagnosed with NIDCM, 12 were excluded due to poor quality of their CMR images, resulting in a final sample size of 98 patients. Basic clinical characteristics, laboratory results, and electrocardiography findings were recorded using a standard format. All participants provided written informed consent prior to enrollment, and the study was approved by the local institutional ethics committee.
- During the study’s second phase, myocardial mechanics and morphometrics were evaluated after 1 year. The study group at this stage consisted of 42 patients, excluding those who had received an implantable device or had passed away. All patients received optimal HF treatment throughout the follow-up period according to established chronic HF guidelines.^11^

The study enrolled a control group of 25 randomly selected individuals without clinical signs of heart disease. These patients underwent 2D echocardiography and CMR, and they also matched the age and gender of the study patients. In this group, no structural heart damage was detected. Parameters of echocardiography and CMR were compared with the study group.

The study was approved by the Kaunas Regional Biomedical Research Ethics Committee (protocol No. BE-2-118 and date of approval: 6 December 2021). All participants provided written informed consent.

### Biomarkers

The following biomarkers were evaluated: troponin I (TnI), CRP, hs-CRP, brain natriuretic peptide (BNP), suppression of tumorigenicity 2 (ST2), and neutrophil to lymphocyte (N/L) ratio. These biomarkers were assessed at the first patient contact and were suspected of the DCM phenotype.

### CMR Protocol and Strain Analysis

CMR examinations were conducted using a 3.0 Tesla MRI system (MAGNETOM Skyra, Siemens Healthcare, Erlangen, Germany), equipped with an 18-channel cardiac coil and electrocardiographic gating. Standard balanced steady-state free precession (bSSFP) sequences were employed to acquire cine images during expiratory breath-hold. This included 2-, 3, and 4-chamber long-axis views and a short-axis stack that covered the entire left and right ventricles. All images were transferred to an offline workstation and analyzed with specialized post-processing software (Medis Suite 3.1, Medis Medical Imaging, Leiden, The Netherlands) by an experienced observer blinded to clinical data.

Short-axis cine images were evaluated using conventional volumetric methods to derive LV and right ventricular (RV) volumes, ejection fractions (LVEF and RVEF), and LV mass.^12^ CMR strain analysis was conducted using dedicated FT software, QStrain (Medis Suite 3.1, Medis Medical Imaging, Leiden, The Netherlands). Endocardial contours of all cardiac chambers were manually delineated at end-systole, followed by automated tracking with manual adjustments applied when necessary.^13^

**Figure 1.**
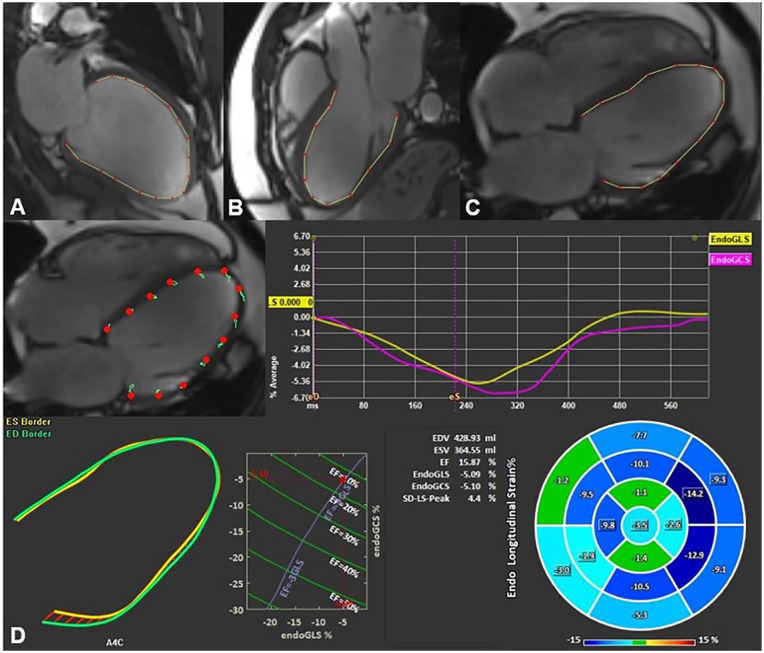
Example of left ventricular global longitudinal strain assessment using feature tracking software. Endocardial borders were delineated on long-axis 2- (A), 3- (B), and 4-chamber (C) SSFP cine images in end-systole and end-diastole. The final automatic calculation was performed by the software: the schematic picture shows minimal myocardium movement and the average GLS of all 17 cardiac segments in this case was −5.09 (D).

Left ventricular global longitudinal strain (LV GLS) and global circumferential strain (GCS) were calculated by averaging peak strain values from a 17-segment model. LV GLS was derived from long-axis cine images in the 2-chamber, 3-chamber, and 4-chamber views (Figure 1).

Mean T1 relaxation times were measured on short-axis T1 maps by manually placing regions of interest (ROIs) in the interventricular septum at the basal, mid, and apical levels of the LV before and after administering a gadolinium-based contrast agent.

Myocardial extracellular volume (ECV) fraction was calculated by placing ROIs centrally within the LV blood pool on native and post-contrast T1 maps, excluding papillary muscles and trabecular structures. Venous blood samples were collected within 24 hours before the CMR examination to determine hematocrit for ECV calculation.

Late gadolinium enhancement (LGE) imaging was performed after intravenous gadobutrol administration (0.1 mmol/kg). An experienced physician visually assessed the images to determine the presence and pattern of enhancement. Only patients demonstrating a non-ischemic LGE pattern were included in the study.

**Figure 2.**
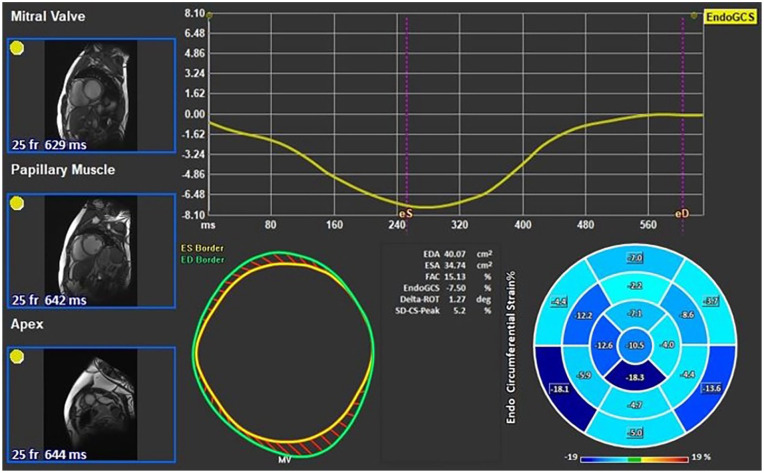
Left ventricular global circumferential strain assessment using feature tracking software.

LV GCS was calculated using previously defined points in QMass on short-axis images at basal, mid-ventricular, and apical levels during both end-systole and end-diastole (Figure 2).

**Figure 3.**
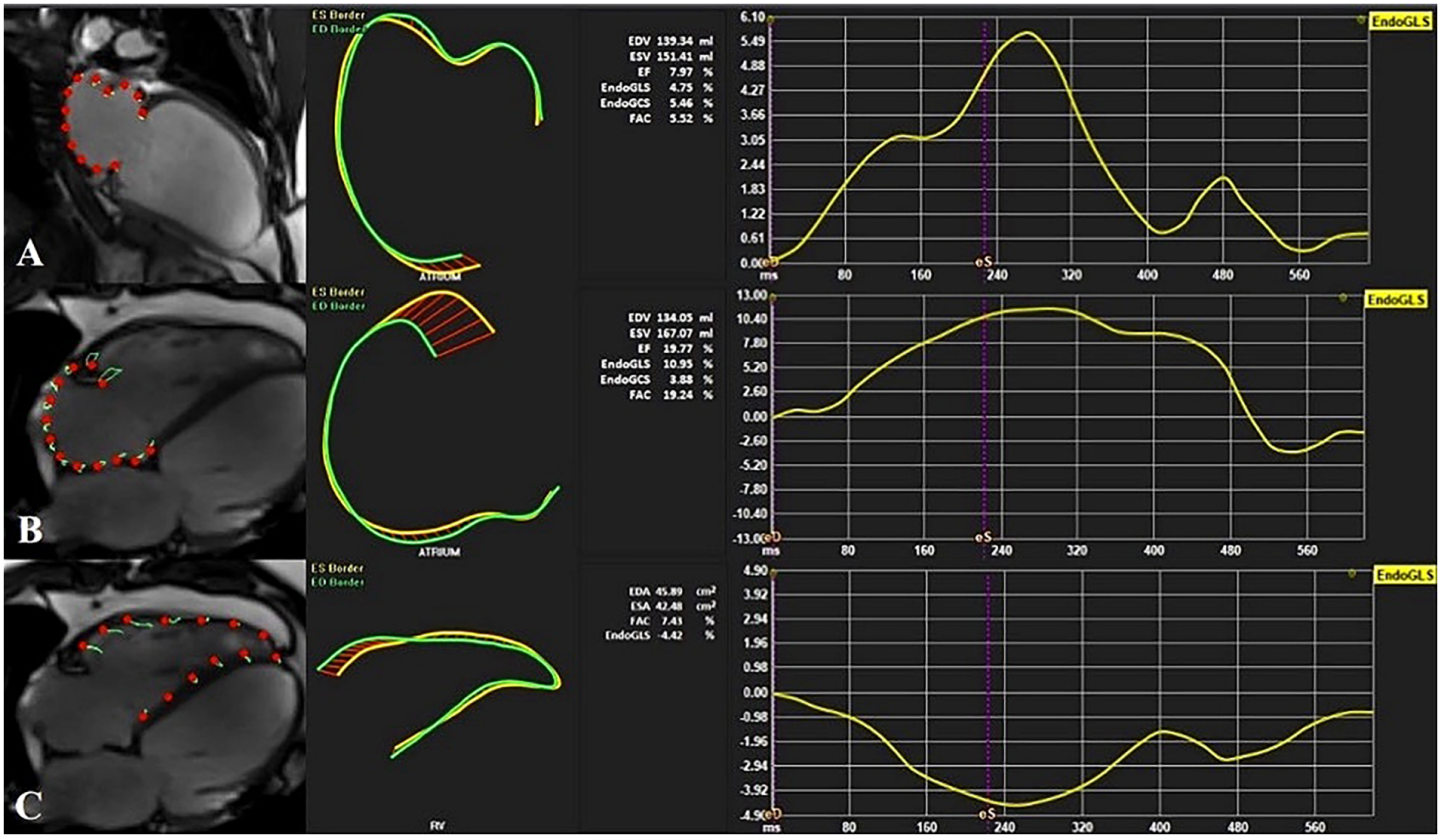
Cardiac magnetic resonance imaging-derived whole-heart myocardial mechanical parameters assessment using feature tracking software.

RV and right atrial (RA) GLS were derived from 4-chamber long-axis images, while LA GLS was assessed from 2-chamber long-axis images (Figure 3).

### Statistical Analysis

The results were expressed as averages ± standard deviations (SD) or total counts and percentages. Participants in the study were divided into 2 groups based on whether they experienced the early primary outcomes. The student’s *t*-test analyzed variables that followed a normal distribution. At the same time, the Mann-Whitney *U*-test was employed for variables that did not meet the criteria for normal distribution. Pearson’s correlation coefficient was utilized to examine the relationships between prognostic factors of HF, myocardial mechanics, and morphometric measurements.

In the multivariate regression analysis, variables that demonstrated a statistically significant association in the univariate analysis (*P* < .05) were initially included. A stepwise regression method was applied, using a removal criterion of *P* > .1. The main variables included in the analysis were TnI, CRP, hs-CRP, BNP, soluble ST2, the N/L ratio, and parameters related to whole heart myocardial deformation and geometry.

Notably, due to the strong association between hs-CRP and LA GLS, a receiver operating characteristic (ROC) analysis was conducted to evaluate the prognostic value of hs-CRP. A limitation of this study is the lack of data from an independent (external) cohort for validating this analysis. However, internal validation using the bootstrap method confirmed the robustness of the findings. These results suggest that hs-CRP may be a valuable prognostic biomarker for identifying patients with declining left atrial mechanics.

The data were analyzed using SPSS version 29 (IBM, Chicago, IL, USA), with a significance level set at a *P*-value of less than .05. Twenty-five patients were randomly evaluated, employing Bland-Altman analysis, to assess intraobserver variability. The results indicated a good level of agreement, with a slight bias of 0.6 ± 2.5%.

## Results

Table 1 presents the demographic and clinical characteristics of the 98 patients with NIDCM who were included in the study. Forty-two of these patients successfully underwent follow-up imaging after 1 year. The limited number of follow-up scans was primarily due to the presence of implanted cardiac devices and the inadequate quality of imaging views in some instances.

**Table 1.** Patient’s Demographic and Clinical Characteristics.

Demographic and clinical variables	NIDCM group (n = 98)
Age, years	49.5 ± 10.1
Male gender, n (%)	68 (69.4)
BMI, kg/m^2^	28.8 ± 5.4
Systolic blood pressure, mmHg	125.9 ± 13.1
Heart rate, bpm	78.5 ± 16.6
Atrial fibrillation, n (%)	37 (37.8)
QRS duration, ms	122.2 ± 30.3
NYHA class, n (%)	
I	2 (2.0)
II	24 (24.5)
III	58 (59.2)
IV	14 (14.3)
Positive family history, n (%)	43 (43.9)
Ventricular tachycardia, n (%)	31 (31.6)
Cardiovascular risk factors, n (%)	
Arterial hypertension	56 (57.1)
Dyslipidemia	38 (38.8)
Obesity	64 (65.3)
Smoker	41 (41.8)
Diabetes	7 (7.1)
Pharmacotherapy (at baseline), n (%)	
ACE-I/ARB	43 (39.4)
Betablocker	51 (46,7)
CCB	28 (25.6)
Aldosterone antagonist	9 (8.2)
Statins	20 (18.3)
Diuretic	3 (2.7)
Laboratory values	
TnI, ng/l	0.3 ± 1.2
CRP, mg/l	9.3 ± 5.9
hs-CRP, mg/l	3.2 ± 2.8
BNP, ng/l	1256.3 ± 680.3
ST2	48.1 ± 8.4
N/L ratio	3.1 ± 3.0

Abbreviations: ACE-I, angiotensin-converting enzyme inhibitor; ARB, angiotensin receptor blocker; BMI, body mass index; BNP, B-type natriuretic peptide; CCB, calcium channel blocker; Hgb, hemoglobin; hs-CRP, high-sensitivity C-reactive protein; N/L, neutrophil to lymphocyte ratio; NIDCM, non-ischemic dilated cardiomyopathy; N/L, neutrophil-to-lymphocyte ratio; NYHA, New York Heart Association; TnI, troponin I.

The average age of participants in the NIDCM group was 49.5 years (±10.1), with a majority being male (68 participants, or 69.4%). The group was considered overweight, with a mean body mass index (BMI) of 28.8 (±5.4). Both the mean systolic blood pressure and heart rate were within normal ranges. Atrial fibrillation was observed in 37.8% of the patients, while ventricular tachycardia was identified in 31.6% of participants. Individuals diagnosed with NIDCM exhibited a broad QRS duration, with approximately 45% showing left bundle branch block. A significant proportion of the patients were classified as belonging to NYHA functional classes III and IV, accounting for 59.2% and 14.3%, respectively. Nearly half (43.9%) of the individuals had a family history of cardiovascular diseases. Each patient with NIDCM presented several risk factors, including arterial hypertension, dyslipidemia, diabetes, smoking, or obesity, with more than half being obese and having hypertension. During the initial consultation, prescribed arterial hypertension medications were typically administered alone or in combination. Additionally, levels of HF biomarkers were found to be elevated.

Table 2 compares CMR results at diagnosis and after 1 year. The control group consisted of 25 patients, and their values were provided alongside those of the study groups. There was a notable decrease in LV size over time, and LV function improved, with LVEF increasing from 29.9 ± 10.7% to 41.3 ± 12.2% (*P* < .001). While there was a tendency for RV size to decrease in the early stages of the disease, this change was not statistically significant (*P* > .05). The size of the LA decreased (33.9 ± 9.7 cm^2^ vs 30.9 ± 11.1 cm,2 *P* = .045), and its function improved (12.5 ± 7.4% vs 15.7 ± 8.2%, *P* = .010) significantly after 1 year.

**Table 2.** Comparison of Cardiac Magnetic Resonance Parameters Between Baseline and 1-Year Follow-Up.

Parameters	Control group (N = 25)	Baseline (N = 98)	After year (N = 42)	*P* value (A vs B)	*P* value (B vs C)	*P* value (A vs C)
LVEDD, mm	54.2 ± 6.3	68.7 ± 13.3	65.6 ± 9.5	<.001	.126	<.001
LVEDDi, mm/m^2^	24.2 ± 5.2	34.3 ± 7.9	31.1 ± 4.5	<.001	<.001	<.001
LVEDV, ml	164.1 ± 39.0	302.8 ± 88.6	258.4 ± 88.5	<.001	<.001	<.001
LVEDVi, ml/m^2^	74.2 ± 24.9	142.4 ± 44.4	121.7 ± 37.7	<.001	<.001	<.001
LVESV, ml	62.7 ± 17.5	212.6 ± 83.8	160.9 ± 85.9	<.001	<.001	<.001
LVESVi, ml/m^2^	28.3 ± 10.3	108.7 ± 40.4	75.5 ± 37.7	<.001	<.001	<.001
LVEF, proc.	61.9 ± 3.8	29.9 ± 10.7	41.3 ± 12.2	<.001	<.001	<.001
RVEDV, ml	161.7 ± 43.5	198.8 ± 54.6	198.6 ± 52.1	<.001	.978	<.001
RVESV, ml	62.5 ± 23.2	111.6 ± 54.6	108.1 ± 52.2	<.001	.619	<.001
LAA, cm^2^	22.4 ± 3.7	33.9 ± 9.7	30.9 ± 11.1	<.001	.045	<.001
RAA, cm^2^	23.2 ± 5.1	27.9 ± 7.1	26.9 ± 7.1	<.001	.272	<.001
LV GLS, proc.	−23.4 ± 3.1	−11.3 ± 4.7	−12.8 ± 5.4	<.001	.020	<.001
LV GCS, proc.	−36.0 ± 4.4	−17.3 ± 7.5	−13.2 ± 7.5	<.001	.103	<.001
RV strain, proc.	−23.9 ± 4.8	−10.8 ± 6.6	−12.8 ± 8.4	<.001	.327	<.001
LA strain, proc.	29.9 ± 8.2	12.5 ± 7.4	15.7 ± 8.2	<.001	.010	<.001
RA strain, proc.	29.3 ± 9.4	16.4 ± 9.1	16.1 ± 8.7	<.001	.878	<.001

Abbreviations: LA, left atrial; LAA, left atrial area; LVEDD, left ventricular end-diastolic diameter; LVEDDi, left ventricular end-diastolic diameter index; LVEDV, left ventricular end-diastolic volume; LVEDVi, left ventricular end-diastolic volume index; LVEF, left ventricular ejection fraction; LVESV, left ventricular end-systolic volume; LVESVi, left ventricular end-systolic volume index; LVGCS, left ventricular global circumferential strain; LVGLS, left ventricular global longitudinal strain; RA, right atrial; RAA, right atrial area; RV, right ventricle; RVEDV, right ventricular end-diastolic volume; RVESV, right ventricular end-systolic volume.

**Figure 4.**
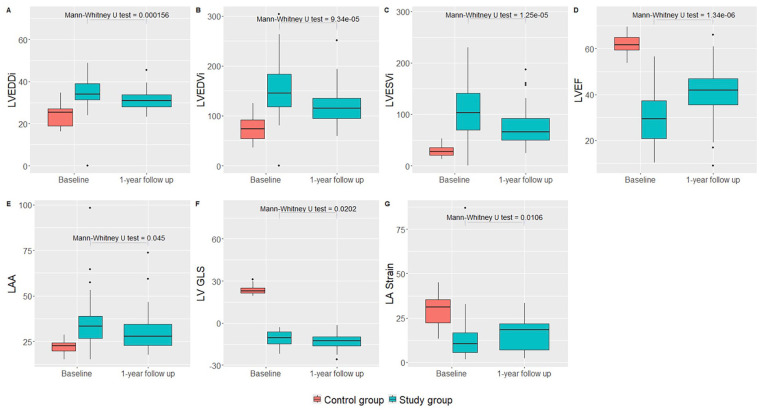
A comparison of cardiac magnetic resonance parameters between different groups. Abbreviations: LA stain, left atrial; LAA, left atrial area; LV GLS, left ventricular global longitudinal strain; LVEDDi, left ventricular end-diastolic diameter index; LVEDVi, left ventricular end-diastolic volume index; LVEF, left ventricular ejection fraction; LVESVi, left ventricular end-systolic volume index.

Figure 4 illustrates the comparison of CMR parameters between the control and study groups. Statistically significant differences were observed in LV end-diastolic diameter index (LVEDDi), LV end-diastolic volume index (LVEDVi), LV end-systolic volume index (LVESVi), LVEF, LA area (LAA), LV GLS, and LA strain (*P* < .05).

Biomarkers’ relationship with whole heart myocardial mechanics and morphometrics was assessed. The strongest correlation was between LA stain and hs-CRP (*r* = −.659, *P* < .001). BNP concentration was weakly correlated with LV size (left ventricular end-systolic volume index (LVESVi) *r* = .280, *P* = .005; left ventricular end-diastolic volume index (LVEDVi) *r* = .334, *P* = .021) and systolic function (LVEF *r* = .387, *P* = .004). N/L ratio was related to changes only in left heart parameters (*P* < .05).

**Figure 5.**
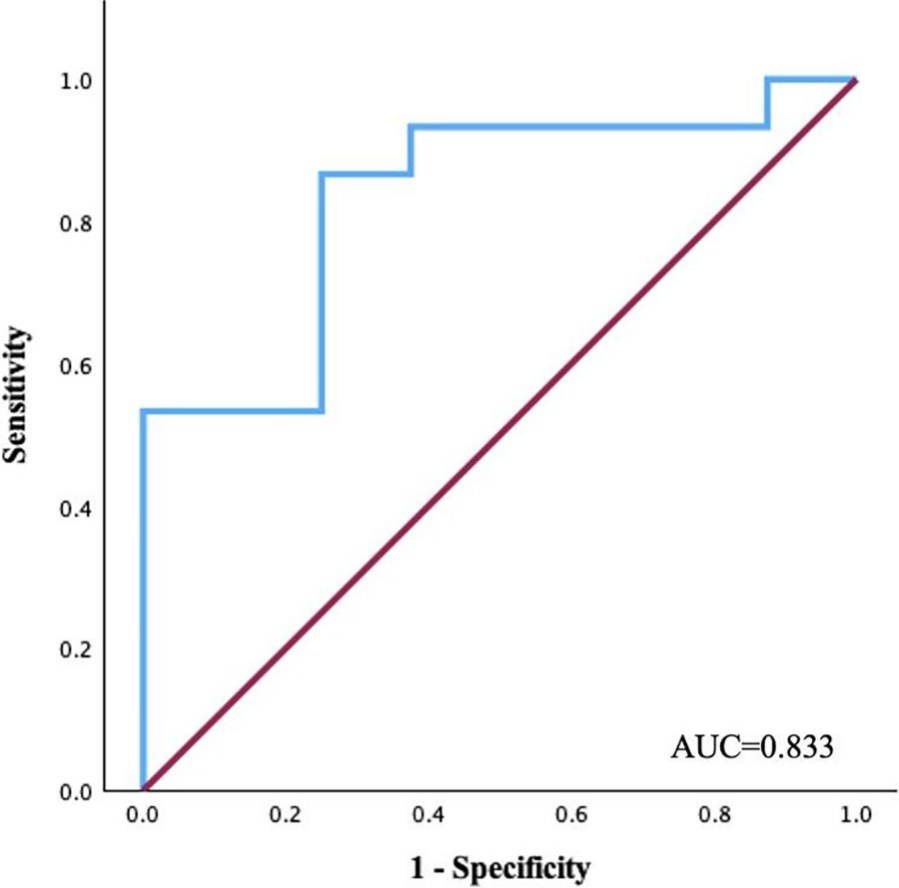
Area under the curve of the model in predicting reduced left atrial global longitudinal strain in patients with NIDCM. Abbreviations: AUC, the area under the curve; red line, reference line.

Since hs-CRP and LA strain had the strongest relationship, ROC analysis was conducted to determine the concentration of hs-CRP that effectively predicts a reduction in LA strain (Figure 5). The cut-off value of the predictive model was defined as the point that yielded the maximum value of the sum of sensitivity and specificity. It was revealed that the cut-off value of 3.6 mg/l of hs-CRP can prognosticate to find a reduced LA GLS with a sensitivity of 100% and specificity of 87% (AUC, 0.833; 95% CI, 0.65–1.008; *P* < .001).

A summarized multivariate regression analysis included the biomarkers for HF prognosis (Table 3). The results indicated that changes in myocardial mechanical parameters and cardiac morphometric parameters after 1 year of follow-up were independently associated with the concentrations of the prognostic biomarkers for HF. The concentration of ST2 was related to early changes in LVESVi with a significance level of *P* < .05. Additionally, the N/L ratio was associated with LV size (*P* = .038). Hs-CRP showed the strongest correlation with LA strain (*P* = .045). BNP levels correlated with changes in LVEF. There wasn’t any relationship between CRP or TnI concentration and myocardial mechanics or morphometric parameters.

**Table 3.** Logistic Regression Analysis for the Biomarkers Related to the Changes in Cardiac Magnetic Resonance Parameters.

Parameter	OR	95% CI	*P*
ST2			
LVESVi, ml/m^2^	−12.875	−26.441-(−1.549)	.030
N/L ratio			
LV GCS, %	−0.325	−0.085-(−0.003)	.038
LAAi, cm/m^2^	−2.180	−1.037-(−0.196)	.007
hs-CRP			
LA GLS, %	−6.169	−10.125-(−0.312)	.045
BNP			
LVEF, %	−0.297	−75.769-(−14.587)	.004

Abbreviations: BNP, brain natriuretic peptide; hs-CRP -high sensitivity C-reactive protein; LA GLS, left atrial global longitudinal strain; LAAi, left atrial area index; LVEF, left ventricular ejection fraction; LV GCS, left ventricular global circumferential strain; LVESVi, left ventricular end-systolic volume index; N/L, Neutrophil-to-lymphocyte ratio; ST2- suppression of tumorigenicity 2.

## Discussion

The findings of this study can be summarized as follows: (1) in patients with NIDCM, the HF biomarkers are most related to changes in left-heart myocardial mechanics and cardiac morphometric parameters; (2) The strongest correlation observed was between hs-CRP and LA GLS.

In this study, we evaluated the early primary outcomes of NIDCM alongside whole-heart myocardial mechanics, cardiac morphometrics, and biomarkers to predict HF prognosis in patients with NIDCM. This study aims to improve the understanding of NIDCM by evaluating the combined effects of myocardial function and geometric changes in all heart chambers.

Over the past years, substantial progress has been made in developing emerging techniques that offer valuable risk prediction, such as genetic factors, imaging, cardiopulmonary exercise testing variables, and circulating biomarkers. While none of the biomarkers are found to be specific to NIDCM, they hold significant potential for risk stratification.^8^ Recently, CMR has become an indispensable cardiac imaging tool, providing detailed, non-invasive insights into both the structure and function of the heart,^5^ including NIDCM.^14^

Previously, in patients with NIDCM, the primary focus was evaluating LV function and morphology.^15^ Recent research has demonstrated that LV GLS is a significant independent predictor of mortality in patients with DCM, providing additional insights beyond CMR parameters like LVEF and LGE.^16^ However, assessing whole-heart myocardial parameters is essential, as the disease affects the LV and cardiac structure.^17-19^ Ventricular dilatation without compensatory wall thickening contributes to impaired wall contractility and diminished cardiac output.^18^ Furthermore, myocardial fibrosis may develop, increasing myocardial stiffness.^20^ Systolic and diastolic dysfunction of LV elevates pressure in LA.^21^ It is important to emphasize that the LA plays a crucial role in overall LV function, particularly in preload and diastolic filling.^21^ In recent years, LA longitudinal strain has been recognized as an independent predictor of prognosis in NIDCM patients with HF.^19,21^ Our study results show that LV GLS and LA longitudinal strain were reduced in the NIDCM group, and a year later, these parameters deteriorated further.

Recent advancements in cardiac imaging, especially with CMR, have shown that RV involvement is common in DCM.^15,22^ RV mechanics generally experience less impairment than LV mechanics in the early stages of dysfunction. This is due to fundamental differences in structure, function, and hemodynamic burden. The right ventricle (RV) operates in the low-pressure pulmonary circuit and relies mainly on longitudinal shortening, which enables it to maintain function longer when faced with chronic volume overload.^15,23^ These insights highlight the importance of accurate and ongoing evaluation of the RV in all DCM patients, with a specific focus on its dynamic behavior throughout the progression of the disease.^15^ Furthermore, studies indicate that right heart involvement is a significant prognostic factor, associated with shorter time to all-cause mortality and an increased risk of ventricular arrhythmic events in HF patients, including those with NIDCM.^24,25^ In conjunction with this, impaired RA function in patients with NIDCM holds significant prognostic value also.^26^ Although the RV and RA function were decreased during diagnosis in our study, these parameters did not change significantly after 1 year, which may be explained by the early disease stage. However, we believe that future studies should include later disease stages. Most studies have focused on changes in individual components of the heart, but the early detection of alterations across all heart myocardial functions and geometry could enhance the accuracy of disease progression predictions and the assessment of sudden cardiac death risk.

As no specific biomarkers exist for NIDCM, this study selected those most practical for clinical assessment and with the most extensive data on their association with HF prognosis.^8^ The relationship between HF and inflammation is bidirectional, functioning both as a cause and a consequence of HF progression and has yet to be fully understood.^27,28^ Recent studies suggest that inflammation and impaired immune system regulation may contribute to DCM pathophysiology, either modulating the disease or as an initiating event (such as acute myocarditis).^29^ It is known that CRP and hs-CRP are nonspecific biomarkers of inflammation associated with endothelial dysfunction.^8^ Several studies have suggested that hs-CRP is an indicator of low-grade inflammation^30^ and is a strong independent predictor of cardiovascular death^31,32^ and poor clinical outcomes in DCM patients.^32,33^ Elevated serum levels of hs-CRP are commonly observed in patients with chronic HF, irrespective of the underlying etiology, especially in those with greater LV systolic dysfunction and more dilated LA and RV.^8^ Although in patients with DCM, the prognostic significance of LGE-CMR has already been documented, studies indicate that LGE-CMR combined with elevated hs-CRP levels is associated with a higher incidence of severe adverse outcomes.^30^ This biomarker is closely linked to myocardial damage, highlighting the connection between early inflammation and ventricular function. It plays a crucial role in ischemic and reperfusion injuries, with hs-CRP serving as a vital biomarker. Its predictive capability offers valuable insights into myocardial injury severity and HF progression in NIDCM patients, aiding clinical decisions and enhancing long-term outcomes. Our study found that changes in hs-CRP levels had the strongest relationship with LA GLS. It can be related to the fact that in DCM, inflammation plays a crucial role as 1 of the initial pathophysiological mechanisms driving early cardiac changes. This inflammatory response can lead to structural and functional alterations within the heart, particularly affecting the LA. In the early stages of the disease, damage to the right side of the heart is typically less severe than that to the left side. As a result, the association with high-sensitivity C-reactive protein (hs-CRP) may be weaker during this time. It is crucial to emphasize the importance of monitoring inflammatory biomarkers, particularly hs-CRP, throughout the entire course of the disease. This includes tracking both its changes over time and the progression of its relationship with alterations in myocardial function and geometry.^34,35,36^ Despite the established link between CRP and worse disease prognosis, we did not observe a significant association between this biomarker and myocardial mechanics or morphometrics. However, this relationship may be revealed in larger-scale studies.

In recent years, the N/L ratio has gained attention as a potential biomarker for predicting disease severity and prognosis in cardiovascular conditions, particularly HF in patients with DCM.^8,37,38^ Systematic reviews and meta-analyses have assessed its prognostic utility in HF.^39^ However, data on the relationship between myocardial mechanics or morphometrics and biomarkers and their impact on prognosis is limited. Our analysis showed that the N/L ratio was associated with changes in left heart parameters, particularly early LV GCS and LA size alterations.

ST2 is a vital biomarker for assessing the prognosis and monitoring of patients with NIDCM. It was thoughtfully included in the 2017 HF guidelines by the American College of Cardiology and the American Heart Association. ST2 is notably reliable across various factors like age, sex, body mass index, renal function, and heart failure history, distinguishing it from BNP and N-terminal pro-hormone BNP (NT-proBNP). While research largely emphasizes its prognostic value, our study explored its link to myocardial mechanics and morphometry, revealing a correlation between ST2 concentration and changes in LV systolic volume.

In response to pressure overload and volume expansion in the ventricles, cardiomyocytes secrete the hormone BNP and its inactive precursor NT-proBNP, due to increased wall stretching.^8,40,41^ BNP and NT-proBNP are widely recognized as key biomarkers in the clinical assessment of HF.^8,41^ Many studies have identified BNP and NT-proBNP as significant prognostic markers in patients with NIDCM.^32,42,43^ Our study uniquely identifies whole-heart myocardial mechanics and geometric parameters that correlate most strongly with BNP. Most studies have shown a relationship between BNP and LV systolic function.^44,45^ Once again, the results of our study confirmed the results of previous studies.

Biomarkers used to evaluate RV dysfunction are mainly derived from the heart and released into the bloodstream due to RV stretch, which are key factors in the development of RV dysfunction. The biomarkers, specifically troponin and BNP, have various applications in RV dysfunction, particularly in pulmonary embolism, where they are crucial for risk stratification and prognosis assessment.^46^ In our study, the correlations between right heart parameters and biomarkers were weak, so only the left heart was included in the multivariate analysis. This may relate to our assessment of correlations between both heart parts and biomarkers. Analyzing only the right heart parameters could reveal stronger correlations. The results suggest that damage to the left heart often occurs earlier and is more significant, leading to stronger biomarker correlations. Future studies should focus on later disease stages, several years after onset.

This study’s data support the idea that CMR imaging is essential in the diagnosis, risk stratification, and management of patients with NIDCM in clinical practice. Speckle-tracking analysis is the preferred method for assessing LA strain parameters. LA deformation is the only CMR parameter that, in addition to systolic function, reflects LV diastolic function. Our study results revealed a strong correlation between hs-CRP and LA GLS. Although serial measurement of hs-CRP is not a part of standard clinical management of NIDCM, it might offer important prognostic and pathophysiological insights. Given that NIDCM is a dynamic disease, repeated hs-CRP measurements might be beneficial to track disease progression, especially as right-sided congestion develops, and serve as a risk stratification biomarker. Elevated hs-CRP levels are a significant warning sign, indicating a likely rapid decline in LA function and an increased risk of arrhythmias. In contrast, lower hs-CRP values suggest a more favorable prognosis, indicating quicker positive remodeling of the LA and an overall improvement in the disease outcomes. This critical understanding of hs-CRP levels provides valuable information for managing DCM and aids in tailoring treatment strategies for better patient outcomes. Therefore, LA GLS and hs-CRP are easy to implement in clinical routine practice. Further research is needed to confirm our findings, which could help manage patients.

## Conclusions

In NIDCM patients, the main biomarkers of HF are more related to left-heart myocardial mechanics and morphometrics. Evaluating the initial level of hs-CRP can help to predict the early changes in LA GLS. Further study is needed to assess these relationships in improving prognosis in patients with NIDCM.

### Limitations

This study has several limitations. We presented single-center results based on a small sample size; a specific sample size calculation was not performed before the study. This cohort’s results don’t necessarily represent all patients with NIDCM. We conducted a short-term follow-up study, which needs further validation by other cohort studies and long-term follow-up. Despite its proven link to poorer disease prognosis, we did not find an association with CRP or TnI. However, this association might be demonstrated in larger-scale studies. We focused solely on baseline biomarkers; however, future studies should emphasize the importance of measuring biomarkers serially over time. Only 42/98 patients had a 1-year follow-up. When many participants drop out or are lost during follow-up, the final sample may not adequately represent the initial population. This can potentially affect the validity of conclusions drawn from the study.

## Abbreviations

BMI, body mass index; BNP, brain natriuretic peptide; CMR, cardiac magnetic resonance; CRP, C-reactive protein; DCM, dilated cardiomyopathy; ECV, extracellular volume; FT, feature tracking; GCS, global circumferential strain; GLS, global longitudinal strain; HF, heart failure; hs-CRP, high-sensitivity C-reactive protein; LA, left atrium; LAA, left atrial area; LGE, late gadolinium enhancement; LV, left ventricle; LVEDDi, left ventricular end-diastolic diameter index; LVEDVi, left ventricular end-diastolic volume index; LVEF, left ventricular ejection fraction; LVESVi, left ventricular end-systolic volume index; NIDCM, Non-ischemic dilated cardiomyopathy; N/L, neutrophil to lymphocyte ratio; NT-proBNP, N-terminal pro-hormone BNP; NYHA, New York Heart Association; RA, right atrium; ROC, receiver operating characteristic; ROI, region of interest; RV, right ventricle; RVEF, right ventricular ejection fraction; SD, standard deviations; ST2, suppression of tumorigenicity 2; TnI, troponin I.
